# Normalization of optical fluence distribution for three-dimensional functional optoacoustic tomography of the breast

**DOI:** 10.1117/1.JBO.27.3.036001

**Published:** 2022-03-16

**Authors:** Seonyeong Park, Frank J. Brooks, Umberto Villa, Richard Su, Mark A. Anastasio, Alexander A. Oraevsky

**Affiliations:** a University of Illinois Urbana–Champaign, Department of Bioengineering, Urbana, Illinois, United States; b Washington University in St. Louis, Department of Electrical and Systems Engineering, St. Louis, Missouri, United States; c TomoWave Laboratories, Houston, Texas, United States

**Keywords:** optoacoustic tomography, photoacoustic computed tomography, breast imaging, functional image, optical fluence estimation, spectral coloring effect

## Abstract

**Significance:**

In three-dimensional (3D) functional optoacoustic tomography (OAT), wavelength-dependent optical attenuation and nonuniform incident optical fluence limit imaging depth and field of view and can hinder accurate estimation of functional quantities, such as the vascular blood oxygenation. These limitations hinder OAT of large objects, such as a human female breast.

**Aim:**

We aim to develop a measurement-data-driven method for normalization of the optical fluence distribution and to investigate blood vasculature detectability and accuracy for estimating vascular blood oxygenation.

**Approach:**

The proposed method is based on reasonable assumptions regarding breast anatomy and optical properties. The nonuniform incident optical fluence is estimated based on the illumination geometry in the OAT system, and the depth-dependent optical attenuation is approximated using Beer–Lambert law.

**Results:**

Numerical studies demonstrated that the proposed method significantly enhanced blood vessel detectability and improved estimation accuracy of the vascular blood oxygenation from multiwavelength OAT measurements, compared with direct application of spectral linear unmixing without optical fluence compensation. Experimental results showed that the proposed method revealed previously invisible structures in regions deeper than 15 mm and/or near the chest wall.

**Conclusions:**

The proposed method provides a straightforward and computationally inexpensive approximation of wavelength-dependent effective optical attenuation and, thus, enables mitigation of the spectral coloring effect in functional 3D OAT imaging.

## 1 Introduction

Optoacoustic tomography (OAT), also known as photoacoustic computed tomography (PACT), is an emerging imaging modality that shows promise in sensitivity for breast cancer detection, especially in dense breasts.^1^^–^^4^ OAT images exhibit greater detection sensitivity for highly vascularized, i.e., aggressive, breast tumors,^5^ and greater diagnostic specificity for all tumors over other modalities, such as x-ray mammography and ultrasound.^1^^,^^5^^,^^6^ Another advantage of OAT over x-ray mammography is that OAT does not involve ionizing radiation.^1^^,^^2^ Due to advances in OAT systems design and image reconstruction, a three-dimensional (3D) volumetric scan of the entire breast is now possible.^3^^,^^5^^,^^7^ At a single near-infrared illumination wavelength, natural chromophores in the breast tissue, such as hemoglobin, act as endogenous OAT contrast agents. From external ultrasound measurements of the pressure induced by the laser pulses, the spatial distribution of the chromophores can be estimated; this provides a qualitative, anatomical measure of the blood vasculature.^3^^,^^5^^,^^7^^–^^10^ Measurements from multiple illumination wavelengths matching the local maximum, minimum, and isosbestic point of deoxy- and oxyhemoglobin can be reconstructed into quantitative estimates of the blood oxygen saturation.^5^^,^^11^^–^^16^ This technology referred to as quantitative OAT (qOAT), also known as quantitative PACT, when combined with ultrasound, provides both anatomical and functional information of the breast that can facilitate detecting tumor angiogenesis and hypoxia.^1^

In 3D OAT breast imaging, it is not feasible to deliver the optical fluence uniformly throughout the whole breast volume, due to optical attenuation in tissue and design constraints of the imaging systems.^5^^–^^7^^,^^9^^,^^13^^,^^17^ Whereas distributions of the optical absorption coefficient are determined by tissue types and physiological status, initial pressure distributions decay with depth in the tissue because of light attenuation. Furthermore, the attenuation is wavelength-dependent. Therefore, direct application of spectral linear unmixing methods to reconstructed OAT images, which correspond to the estimated initial pressure distributions, results in inaccurate estimates of blood oxygen saturation.^14^^,^^15^^,^^18^^,^^19^

To improve visualization of the reconstructed volumetric images, the optoacoustic imaging community has utilized a commercial tool, AMIRA (Thermo Fisher Scientific),^20^ and open source, interactive tools such as ImageJ (Wayne Rasband),^21^ ParaView (Kitware),^22^ and 3D Slicer (Kitware).^23^ Also, a 3D PHOVIS (POSTECH, Korea)^24^ has been recently released that is developed specifically for optoacoustic imaging. However, these tools do not provide physics-informed image processing for contrast enhancement at depth in reconstructed OAT volumes. In addition, these visualization tools are semi-automatic and require substantial manual intervention by the user. Pattyn et al.^25^ proposed a model-based method to compensate for the optical fluence distribution within a heterogeneous physical phantom that mimics a breast. Monte Carlo (MC) simulation was employed, and known optical properties of the phantom were assumed. However, in practice, the distributions of optical properties within the breast are generally unknown.

A straightforward physics-informed image processing method is proposed to compensate for both the nonuniform incident optical fluence at the breast surface and the wavelength- and depth-dependent optical attenuation within the breast, and the impact of the proposed method on accuracy of the linear unmixing of deoxy- and oxyhemoglobin is investigated. The contributions of this paper are twofold. First, this study establishes an implementation of the linear unmixing method for use with a large object such as a female breast. Second, the proposed method improves sensitivity of the 3D OAT breast imaging by improving contrast at depth.

The paper is organized as follows. Background materials, including existing illumination systems in 3D OAT breast imaging and spectral linear unmixing, are provided in Sec. 2. The proposed method is explained in Sec. 3, and the study description and evaluation metrics are provided in Sec. 4. Results from computer-simulation and experimental studies are presented in Sec. 5, and a discussion is given in Sec. 6. The conclusions of the study are provided in Sec. 7.

## 2 Background

In OAT imaging, a short laser pulse is employed to irradiate an object at time t=0 and conversion of the absorbed optical energy into the thermal energy results in the generation of an initial pressure distribution $p_{0}(r,\lambda )$, where $r=(x,y,z)\in R^{3}$ and λ is a wavelength. The pressure distribution subsequently propagates and is measured by multiple ultrasonic transducers located on a measurement aperture $\Omega_{0}\subset R^{3}$ that partially or completely surrounds the object. The propagated pressure wavefields, i.e., the optoacoustic signals, at time t>0 are denoted as p(r,t).

By solving the associated acoustic inverse problem,^26^ an estimate of the absorbed energy density distribution within the object can be obtained. Functional quantities such as the vascular blood oxygenation can be reconstructed via qOAT from multiwavelength measurements.^5^^,^^11^^–^^15^^,^^27^

In most implementations of 3D OAT breast imaging, the patient lies prone on a bed with their breast located inside a water-filled bowl just below the surface plane of the bed. The breast is illuminated at a near-infrared wavelength with a short laser pulse.^3^^,^^5^^,^^7^^,^^9^^,^^17^^,^^28^ The induced pressure waves p(r,t) propagate out of the breast and are measured with the ultrawide-band ultrasonic transducers.^16^

### 2.1 Illumination in 3D OAT Breast Imaging

Several different 3D OAT breast imaging systems have been proposed and established, but their data-acquisition principles are similar.^3^^,^^5^^,^^7^^,^^9^^,^^17^^,^^28^ Existing systems for 3D OAT breast imaging are equipped with two subsystems: an illumination system and an optoacoustic signal detection system. The focus here is on the illumination system. A common feature in the existing illumination systems is that the light is delivered in a radially symmetric pattern to the breast surface from either single^3^^,^^7^^,^^9^^,^^17^^,^^28^ or multiple^5^ light sources. The laser fluences involved are well below the maximum permissible exposure for skin defined by the American National Standards Institute (ANSI).^29^

**Fig. 1:**
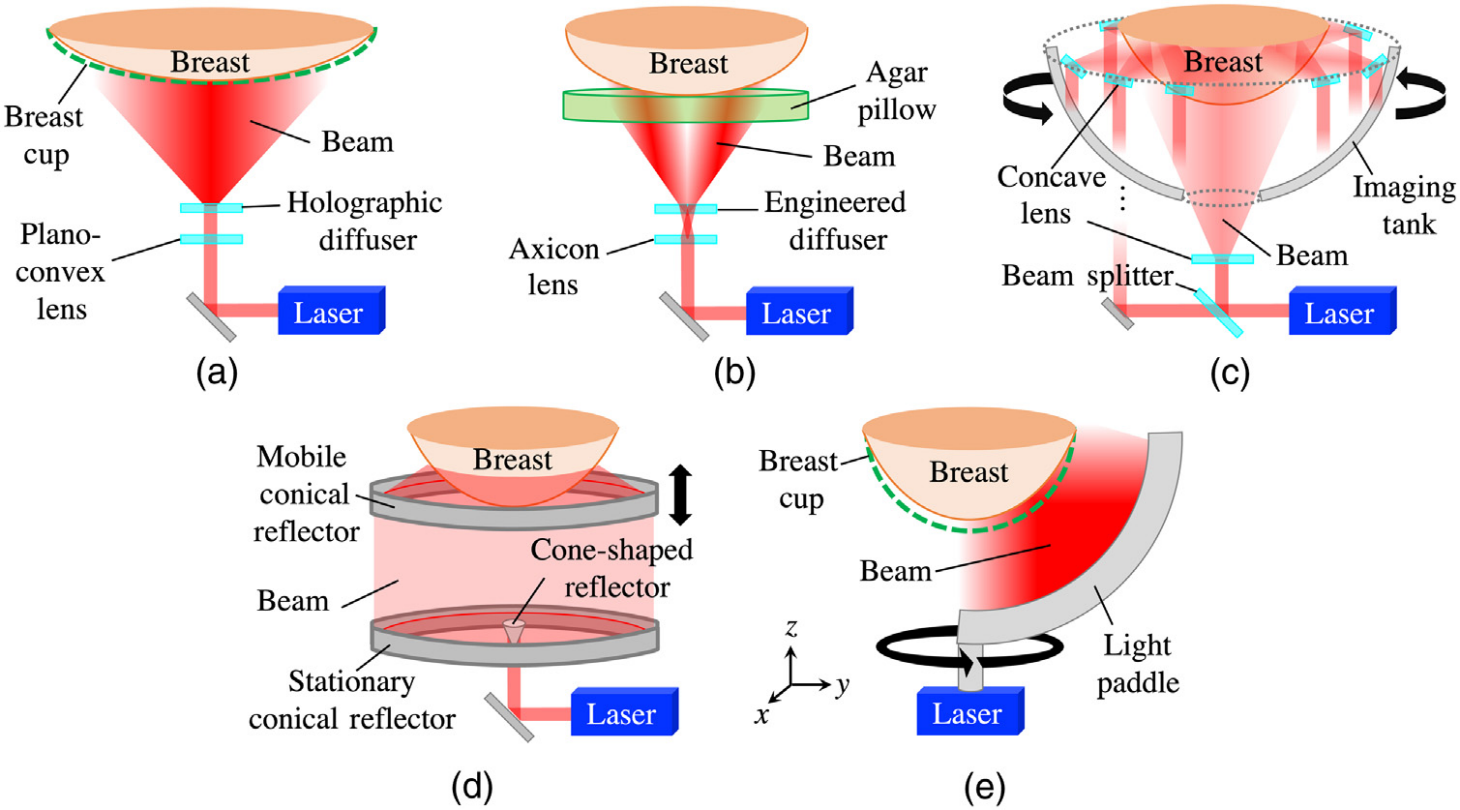
Illumination in 3D OAT imaging systems for the breast: (a) Kruger et al.^9^ and Toi et al.,^7^ (b) Lin et al.,^3^ (c) Schoustra et al.,^28^ (d) Alshahrani et al.,^17^ and (e) Oraevsky et al.^5^

Table 1 presents salient design features of the light-delivery systems employed by the five OAT breast imaging systems shown in Fig. 1. These systems deliver the light to the entire breast surface. However, less optical energy per unit area (optical fluence) is delivered to regions near the chest wall compared with the center of the breast, and this imbalanced distribution of the incident optical fluence causes lower voxel brightness near the chest wall in the reconstructed OAT images.

**Table 1:** Light-delivery systems employed by the five OAT breast imaging systems in Fig. 1.

Ref.	λ (nm)	Salient design features
7,9	755, 795	• A conical laser beam is emitted from below and then reflected through a planoconvex lens and a holographic diffuser [Fig. 1(a)].
		• The breast is contained by a 0.5-mm-thick polyethylene terephthalate glycol-modified (PETG) cup which is optically and acoustically transparent.
3	1064	• A donut-shaped laser beam is emitted from below and then reflected through an axicon lens and an engineered diffuser [Fig. 1(b)].
		• An agar pillow is used to slightly compress the breast.
28	755, 1064	• A laser beam is split into a bottom beam and side beams, and then those are diverged via concave lenses [Fig. 1(c)].
		• The side beams are emitted slightly upward from nine optical fiber bundles that rotate around the breast in discrete steps.
17	532	• A ring-shaped light beam is formed via a cone-shaped reflector, stationary conical reflector, and mobile conical reflector [Fig. 1(d)].
		• The mobile conical reflector vertically moves together with the ring-shaped detector array during the scan.
5	755 to 1064	• Laser beams are emitted from five fiber-optic segments that are constrained to the surface of a light paddle that rotates around the breast in discrete steps [Fig. 1(e)].
		• The PETG breast cup is 0.1 mm thick.

### 2.2 Spectral Linear Unmixing

Several qOAT methods have been proposed to estimate the optical absorption coefficient $\mu_{a}(r,\lambda )$ and/or oxygen saturation distribution.^13^^–^^15^^,^^30^^–^^37^ Among them, a two-step spectral linear unmixing approach has been widely used.^5^^,^^13^^,^^18^^,^^30^^–^^36^ The first step of the method is OAT image reconstruction (i.e., acoustic inversion) to estimate the initial pressure distribution $p_{0}(r,\lambda )$ that is induced via the optical absorption and subsequent nonradiative relaxation of electronic energy into heat. The second step is to approximate the oxygen saturation distribution from the estimates of $p_{0}(r,\lambda_{i})=\Gamma \mu_{a}(r,\lambda_{i})\phi (r,\lambda_{i})$^11^^,^^38^ acquired at multiple wavelengths (i=1,…,n,n∈N). Here, Γ is the dimensionless Grüneisen parameter that can be considered constant for soft tissues.^11^^,^^38^^,^^39^

In unmixing methods, reconstructed estimates of $p_{0}(r,\lambda_{i})$ are considered as surrogates of $\mu_{a}(r,\lambda_{i})$. Unmixing procedures are predicated upon the relationship $\mu_{a}(r,\lambda_{i})=\epsilon_{\mathrm{Hb}}(\lambda_{i})C_{\mathrm{Hb}}(r)+\epsilon_{{\mathrm{HbO}}_{2}}(\lambda_{i})C_{{\mathrm{HbO}}_{2}}(r)$, where $\epsilon_{\mathrm{Hb}}$ and $\epsilon_{{\mathrm{HbO}}_{2}}$ are known wavelength-dependent molar extinction coefficients and $C_{\mathrm{Hb}}$ and $C_{{\mathrm{HbO}}_{2}}$ denote molar concentrations of deoxy- and oxyhemoglobin, respectively.^11^^–^^13^^,^^18^ The molar concentrations of $C_{\mathrm{Hb}}(r)$ and $C_{{\mathrm{HbO}}_{2}}(r)$ are computed as

$$[\begin{array}{c} C_{\mathrm{Hb}}(r)\\ C_{{\mathrm{HbO}}_{2}}(r) \end{array}]=\epsilon^{+}[\begin{array}{c} \mu_{a}(r,\lambda_{1})\\ \vdots \\ \mu_{a}(r,\lambda_{n}) \end{array}],\;\epsilon \equiv [\begin{array}{cc} \epsilon_{\mathrm{Hb}}(\lambda_{1}) & \epsilon_{{\mathrm{HbO}}_{2}}(\lambda_{1})\\ \vdots & \vdots \\ \epsilon_{\mathrm{Hb}}(\lambda_{n}) & \epsilon_{{\mathrm{HbO}}_{2}}(\lambda_{n}) \end{array}],\;\text{and }n\geq 2,$$ (1)

where $\epsilon^{+}$ is a pseudoinverse of the molar extinction coefficient matrix ϵ. Given $C_{\mathrm{Hb}}(r)$ and $C_{{\mathrm{HbO}}_{2}}(r)$, the oxygen saturation distribution is computed as $sO_{2}(r)=\frac{C_{{\mathrm{HbO}}_{2}}(r)}{C_{\mathrm{Hb}}(r)+C_{{\mathrm{HbO}}_{2}}(r)}\times 100\%$. The distribution of the total hemoglobin concentration is calculated as $C_{t\mathrm{Hb}}(r)=C_{\mathrm{Hb}}(r)+C_{{\mathrm{HbO}}_{2}}(r)$, and subsequently the blood vasculature can be detected.

In practice, the distribution of the optical fluence ϕ(r,λ) in breast tissues is not constant because of nonuniform illumination and optical attenuation. Once the light reaches the breast, the optical fluence decreases approximately exponentially with depth; this is described by the well-known Beer–Lambert law: $\phi (d,\lambda )=\phi_{0}\;\exp(-\mu_{\mathrm{eff}}(\lambda )d)$. Here, ϕ(d,λ) denotes optical fluence at a depth d and a wavelength λ, $\phi_{0}$ is the incident optical fluence to the breast surface (d=0), and $\mu_{\mathrm{eff}}(\lambda )$ is an effective attenuation coefficient at the wavelength of λ that reflects both the scattering and absorption of light in tissues.^6^^,^^12^^,^^40^ In addition, it is challenging to uniformly deliver the light to the breast surface in 3D OAT imaging. Hence, the reconstructed $p_{0}(r,\lambda )$ exhibits undesirable variations in the voxel brightness according to design of the illumination system.^5^^,^^6^ This limits the visible depth and field of view in the reconstructed $p_{0}(r,\lambda )$.

Most significantly, the effective attenuation coefficient $\mu_{\mathrm{eff}}(\lambda )$ is wavelength-dependent, which is known as the “spectral coloring effect.”^41^ Thus, in the linear unmixing employing multiple wavelength estimates of $p_{0}(r,\lambda )$ as surrogates of $\mu_{a}(r,\lambda )$, the oxygen saturation distribution cannot be accurately estimated without compensation for $\mu_{\mathrm{eff}}(\lambda )$.^14^^,^^15^^,^^19^^,^^42^ Besides, $p_{0}(r,\lambda )$ is exponentially attenuated with depth, so the compensation for $\mu_{\mathrm{eff}}(\lambda )$ is more important for large organs such as a female breast, that usually has the maximum depth larger than 20 mm, compared to relatively small organs such as skin with the maximum illumination depth of just a few millimeters.

## 3 Normalization of Optical Fluence Distribution

**Fig. 2:**
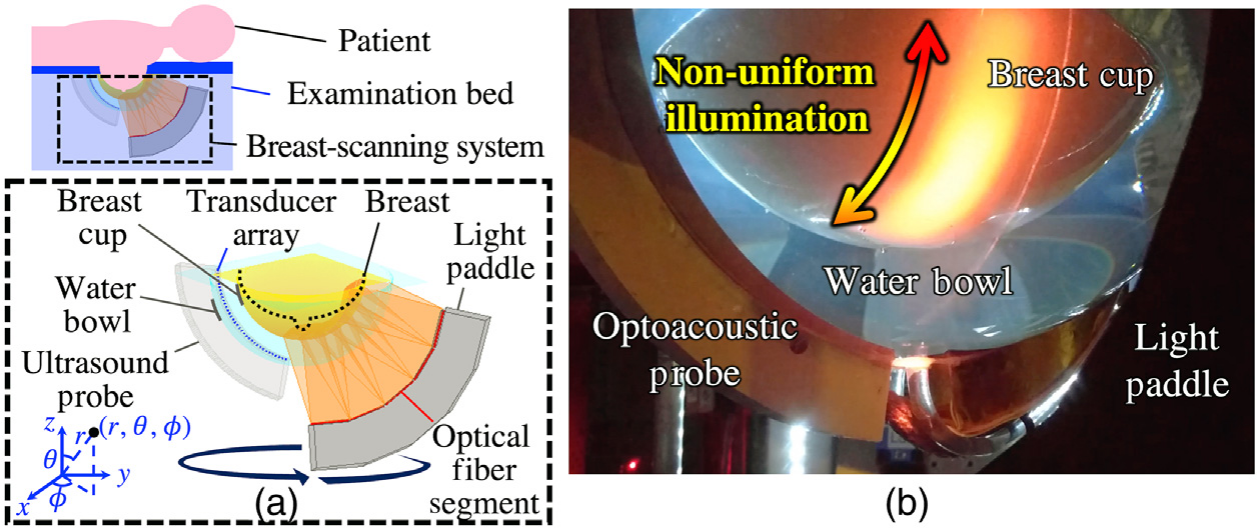
3D OAT scan using LOUISA-3D: (a) breast scan schematic and (b) photograph of phantom scan. (b) Nonuniform illumination is observed on the surface of the tissue-mimicking physical phantom.

**Fig. 3:**
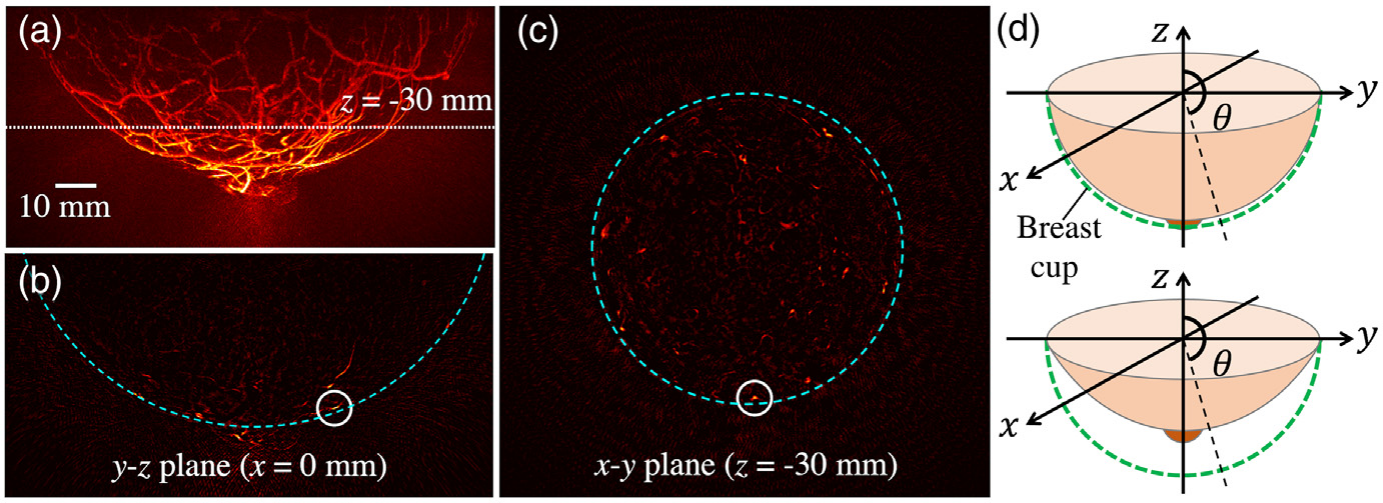
(a)–(c) 3D OAT breast image ($\hat{\alpha }$) of a healthy volunteer at a wavelength of 755 nm, scanned by TomoWave Laboratories using LOUISA-3D^5^ at MD Anderson Cancer Center, and (d) breast shapes in a given breast cup. Maximum voxel brightness projection (MVBP) along (a) x axis and cross-sections on (b) y-z plane at x=0 mm and (c) x-y plane at z=−30  mm. The slice is indicated with a white dotted line in (a). The image ($\hat{\alpha }$) was reconstructed using filtered backprojection (FBP)^43^ method. The brightness range of the images was adjusted for better visibility. In panels (b) and (c), a white solid circle indicates the location of the brightest voxel in the cross-section, and a cyan dashed line represents the approximated breast boundary.

The proposed method seeks to estimate and compensate for the nonuniform optical fluence distribution. This is referred as normalization of the optical fluence distribution hereafter. The method was designed based on a common feature of the existing illumination systems; specifically, that radially symmetric light delivery to the breast surface is employed. In this section, the details of the method are described based on the reference imaging system shown in Fig. 2, where a patient lies prone on the examination bed and the patient’s breast is located inside the breast cup. A spherical coordinate system is assumed, with the origin corresponding to the center of the breast cup [see Figs. 2(a) and 3(d)]. Here, θ is defined to be a polar angle from the positive z axis with 0≤θ≤π.

In the proposed method, the nonuniform distribution of the optical fluence within the breast is estimated from the voxel values in the reconstructed 3D OAT image α^ that is an estimate of $p_{0}(r,\lambda )$ discretized employing a uniform Cartesian lattice. The following reasonable assumptions are made:

A1. The distribution of the incident optical fluence varies along the polar direction (θ) and is radially symmetric on x-y planes [see Fig. 3(a)];
A2. Blood vessels absorb more optical energy than other breast tissues do because deoxy- and oxyhemoglobin of red blood cells are the only optically absorbing chromophores at near-infrared wavelengths that are typically used in OAT breast imaging. Moreover, for wavelengths near 800 nm, artery and vein are nearly indistinguishable from each other in the reconstructed image α^^12^^,^^44^ [see Figs. 3(a)–3(c)];
A3. Anatomically, at least one voxel corresponding to a blood vessel near the skin layer exists at any polar angle in the reconstructed image α^ [see Figs. 3(a)–3(c)];
A4. The shape of the breast located inside a hemispherical stabilizer cup is a partial spheroid and static [see Fig. 3(d)];
A5. The Beer–Lambert law^6^^,^^12^^,^^40^ can be used to approximate the optical fluence distribution within the breast.

The normalization of the optical fluence distribution is conducted in the following order: (1) compensation for nonuniform incident optical fluence, (2) estimation of breast surface and depth of each voxel relative to the breast surface, and (3) compensation for the effective optical attenuation. The location of the breast surface must be known for optical attenuation compensation, but breast surface detection is highly challenging because the top skin layer (epidermis) can appear dimmer than the noise surrounding the breast due to the nonuniform incident optical fluence [see Figs. 3(a)–3(c)]. Therefore, the incident optical fluence needs to be normalized in advance of the breast surface detection.

In the proposed method, a hemispherical breast stabilizer cup is assumed, as it is employed in several 3D OAT breast imaging systems.^5^^,^^7^^,^^9^ The cup is selected for each breast size, so it maintains the breast shape radially symmetric. Therefore, the only possible breast shape is a partial spheroid, as shown in Fig. 3(d). Whereas the nipple and areola absorb more light than the other breast tissues due to their high concentration of pigment, their impact on the optical fluence distribution within the breast is insignificant because of their relatively small volume.^45^ Thus, in the proposed method, the region 160  deg<θ≤180  deg, which was determined by an average diameter ratio of the breast and areola,^45^ is excluded from consideration.

**Fig. 4:**
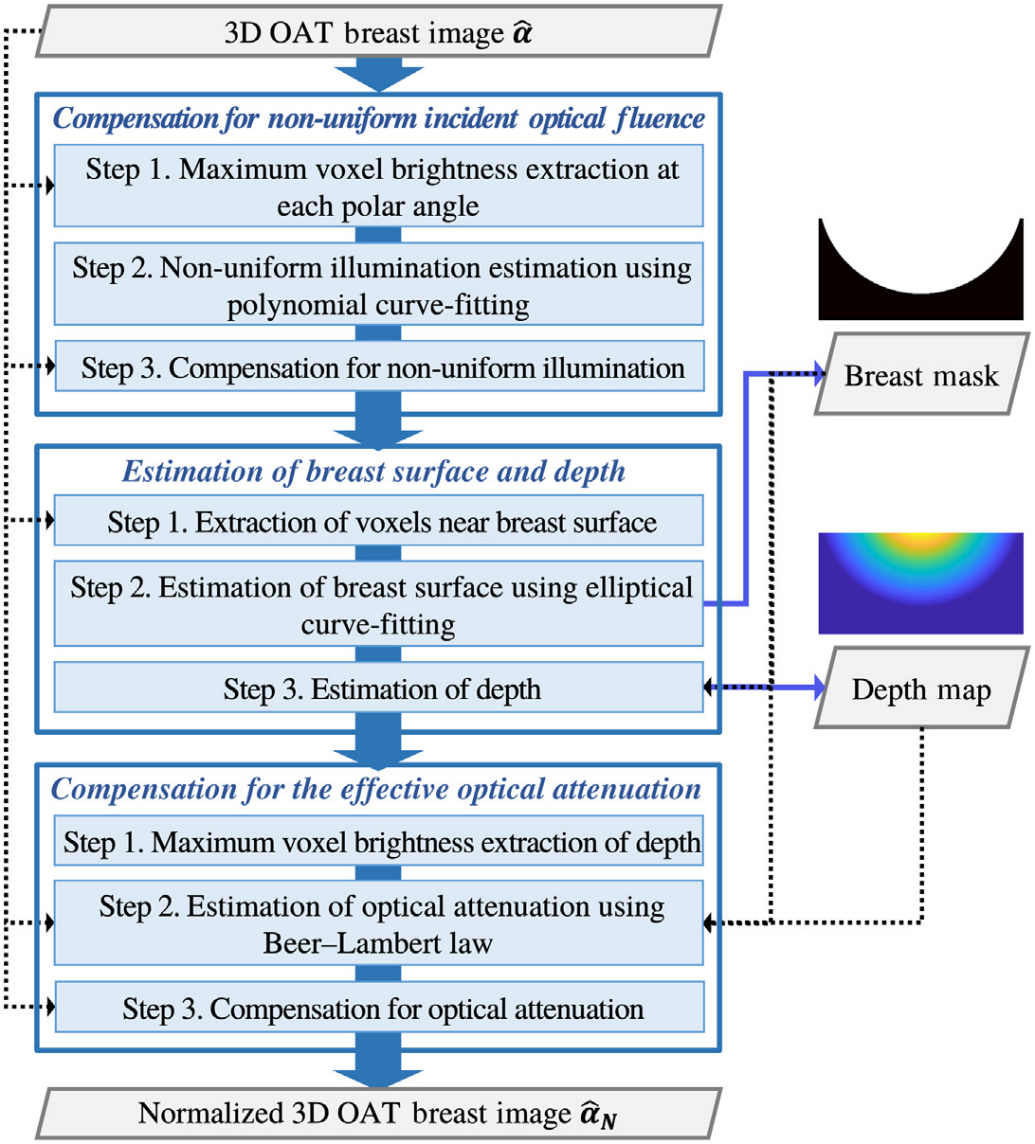
Flowchart of normalization of optical fluence distribution in 3D OAT breast images.

The flowchart of the proposed method is provided in Fig. 4, and the details of each step are given in the following sections.

### 3.1 Compensation for Nonuniform Incident Optical Fluence

Under assumption A1, the distribution of the incident optical fluence is radially symmetric, which means it can be interpreted as a function of polar angle [Fig. 3(d)]. If strong optical absorbers that have similar $\mu_{a}$ values, such as blood vessels, are densely located near the object surface (d=0), the spatial distribution of $p_{0}$ on the surface is proportional to the distribution of the incident optical fluence ($p_{0}\propto \phi$). As mentioned earlier, the blood vessels in subdermal regions appear as the brightest voxels at any polar angle in the reconstructed image α^ [assumption A3; see Figs. 3(b) and 3(c)]. Thus, the distribution of the incident optical fluence can be approximated by the voxel brightness of the blood vessels near the breast skin layer (subdermal), i.e., maximum voxel brightness projection (MVBP) at discretized polar angles $\left[\theta {]}_{n}\in (\theta_{i}-\frac{\Delta \theta }{2},\theta_{i}+\frac{\Delta \theta }{2}\right)$ with an increment Δθ of 1 deg. The polar angle $[\theta {]}_{n}$ is calculated as $\cos^{-1}(z_{n}/r_{n})$, where $r_{n}=\sqrt{x_{n}^{2}+y_{n}^{2}+z_{n}^{2}}$ is the distance of the *n*’th voxel from the origin, and $x_{n}$, $y_{n}$, and $z_{n}$ are the x, y, and z-coordinates of the *n*’th voxel in the uniform Cartesian grid, respectively.

**Fig. 5:**
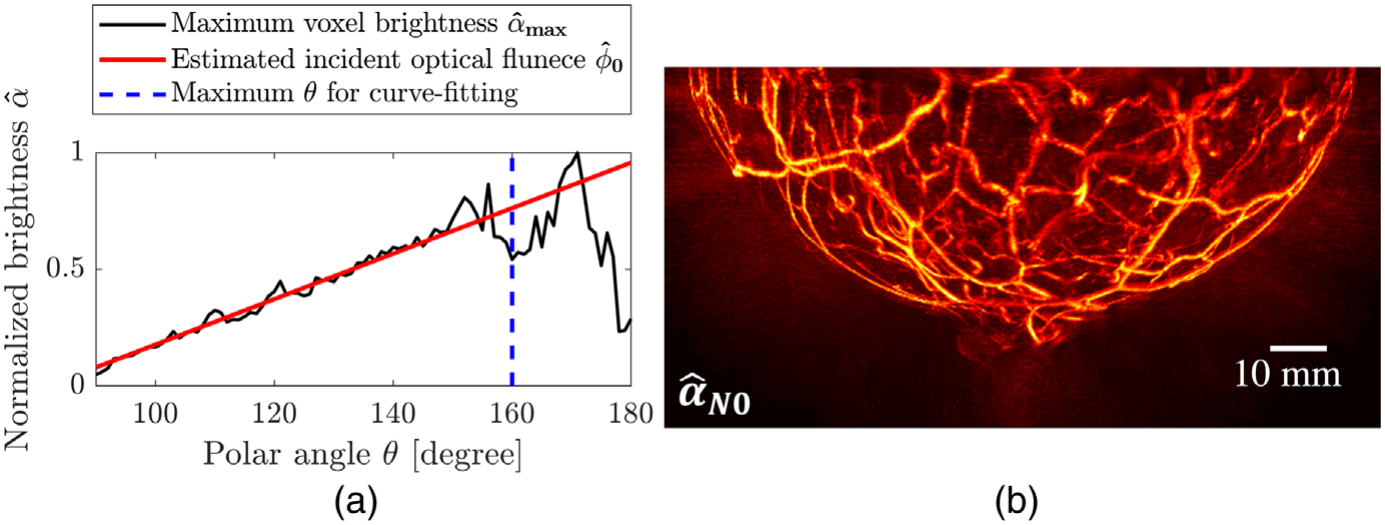
Compensation for nonuniform incident optical fluence: (a) estimated incident optical fluence as a function of polar angle and (b) MVBP of 3D OAT breast image after the compensation (α^N0) along x axis. The results were obtained from Fig. 3(a). In panel (a), a black solid line indicates the maximum voxel brightness according to polar angles, and a red solid line represents a first-degree polynomial curve $q_{1}({\left[\theta \right]}_{i})$ fitted to the maximum voxel brightness. The polar angles to the right of the blue dashed line correspond with nipple and areola in (a).

An L-degree polynomial curve $q_{L}(\theta )$ is fitted to the extracted maximum voxel brightness according to the discretized polar angles within the range of interest (90 deg to 160 deg), where the region containing the nipple and areola is excluded, as shown in Fig. 5(a). The estimated distribution of the incident optical fluence ϕ^0 is described as

[ϕ^0]n=qL([θ]n),n=1,…N, (2)

where N is the total number of voxels. The L is set depending on the illumination pattern. It was found that L=1 and L=2 were sufficient for accurately fitting the data in the experimental studies (Sec. 4.2) and computer-simulation studies (Sec. 4.1), respectively.

Elementwise division of ϕ^0 is applied to the reconstructed image α^, to compensate for the nonuniform incident optical fluence as shown in Fig. 5(b):

[α^N0]n=[α^]n[ϕ^0]n,n=1,…,N. (3)

**Fig. 6:**
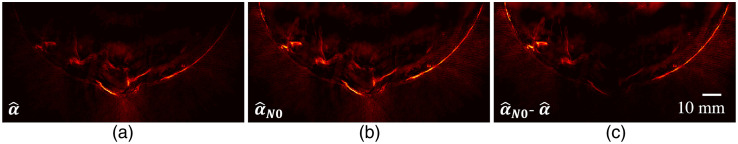
Comparison of images before and after compensation for non-uniform incident optical fluence: MVBP of a 5-mm-thick y slice at y=0 mm (a) before (α^) and (b) after (α^N0) the compensation along y axis and (c) MVBP of their difference (α^N0−α^) along y axis. The results were obtained from Fig. 3(a). The voxel brightness near the chest wall (θ=90  deg) in (a) is lower than in the region near the areola (θ≥160  deg) and, accordingly, the compensation procedure leads to a higher gain near the chest wall as shown in (b) and (c).

A comparison of the images before and after the compensation is shown in Fig. 6

### 3.2 Estimation of Breast Surface and Depth

Under assumption A3, the blood vessels located in subdermal regions can be employed to infer the breast surface in the reconstructed 3D OAT image α^. The average range of skin thickness of healthy female human breasts is between 0.5 and 2.4 mm.^46^ This is a relatively thin layer that attenuates light negligibly in comparison to attenuation in the bulk. To extract the voxels that belong to blood vessels in the close proximity of the breast surface, first a 3D median filter is applied to reduce the noise. Subsequently, the contrast of the resulting image is increased by elementwise square operation:

[α^N0′]n=([med{α^N0}]n)2,n=1,…,N, (4)

where med{·} is a 3D median filter function with a 3-by-3-by-3 filter.

The voxels corresponding to the blood vessels near the breast surface are extracted using Otsu thresholding^47^ applied to α^N0′. The set of the extracted voxels is defined as V={n≤N:[α^N0′]n≥T} where T is an Otsu’s threshold. For each polar angle, the voxels in V that are the farthest from the z axis are selected to estimate the breast boundary. The estimated radius of the breast in the cross-section of slice (j’th z-slice), is given as

$$[\rho_{z}{]}_{j}=\max\{[\rho {]}_{n}\}\;n\in S_{j}\cap V,$$ (5)

where $[\rho {]}_{n}$ is the distance of the n’th voxel from the z-axis calculated as $\sqrt{x_{n}^{2}+y_{n}^{2}}$, and $S_{j}$ denotes the set of all voxels, on the j’th z-slice, i.e., the x-y plane.

**Fig. 7:**
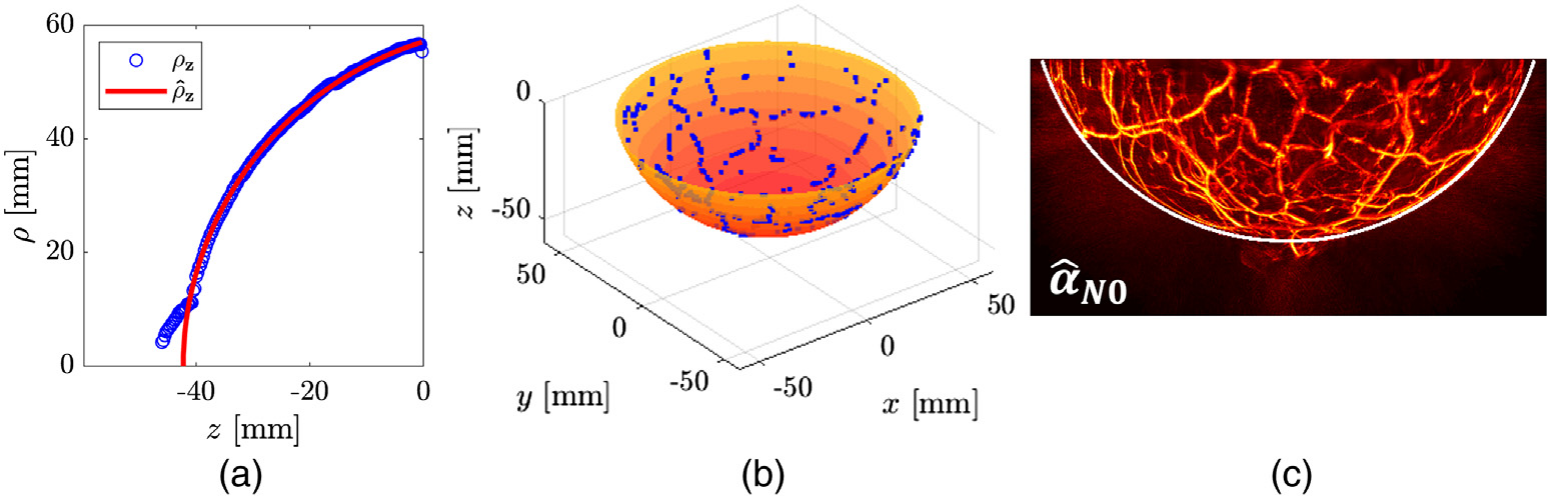
Breast surface estimation: (a) estimated radii on x-y planes ρ^z; (b) estimated breast surface; and (c) estimated breast boundary on y-z plane at x=0 overlaid on the MVBP of α^N0 along the x axis.

In Fig. 7(a), blue circle markers correspond to an estimate $\rho_{z}$ for each z-slice. An elliptical curve is fit to $\rho_{z}$ to obtain a smooth representation of the breast boundary according to assumption A4. The ellipse equation is $\frac{(z-z_{C}{)}^{2}}{a^{2}}+\frac{\rho^{2}}{b^{2}}=1$. Here, a and b are lengths along semimajor and semiminor axes, respectively, $z_{C}$ is a z-coordinate of the ellipse center. These parameters are determined by the elliptical curve fitting. The surface formed by rotation of the estimated elliptical curve [a red solid line in Fig. 7(a)] around the z axis is then used to approximate the breast surface as shown in Fig. 7(b). From this, a breast mask $M_{\text{Breast}}$ is built by assigning the value “1” to voxels inside the surface and “0” outside.

Finally, the depth d at each voxel that is used in the Beer–Lambert law is approximated as the minimum distance from the breast surface, i.e., the estimated spheroid surface, using Newton’s method.^48^

### 3.3 Compensation for the Effective Optical Attenuation

With consideration of assumptions A2 and A5, optical attenuation can be approximated as a function of depth from the decay of the voxel brightness inside the blood vessels in the reconstructed 3D OAT image. Specifically, such an approximation uses the maximum voxel brightness value in a certain depth range of $\left(d_{m}-\frac{\Delta_{d}}{2},d_{m}+\frac{\Delta_{d}}{2}\right)$, where $\Delta_{d}$ is an increment of 1 voxel:

[α^BV]m=maxn∈Cm{[α^]n}. (6)

**Fig. 8:**
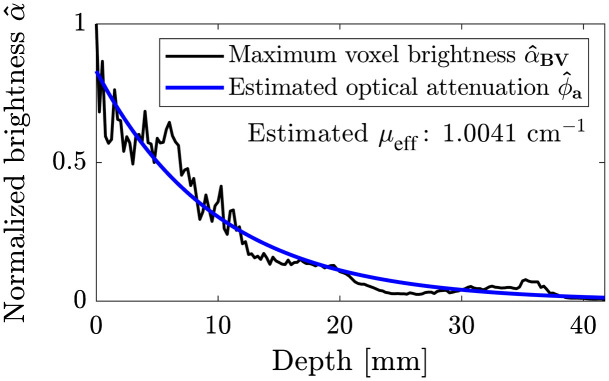
Estimation of optical attenuation at a wavelength of 755 nm. A black solid line indicates maximum voxel brightness α^BV at a certain depth range of $\left(d_{m}-\frac{\Delta_{d}}{2},d_{m}+\frac{\Delta_{d}}{2}\right)$, and a blue curve is the estimated optical attenuation ϕ^a. The $\mu_{\mathrm{eff}}$ estimated from the 3D OAT breast image was $1.0041\;{\mathrm{cm}}^{-1}$.

Here, $C_{m}$ denotes a set of voxels in the m’th depth range. Figure 8 shows α^BV according to depth.

An exponential curve based on the Beer–Lambert law (assumption A5) is fit to α^BV as shown in Fig. 8. The estimated optical attenuation is expressed as

[f^a]n=c* exp(−μeff*[d]n),n=1,…,N, (7)

where $c^{*}$ and $\mu_{\mathrm{eff}}^{*}$ are the prefactor and the effective optical attenuation coefficient estimated from the curve fitting, respectively.

Elementwise division of ϕ^a is applied to the image after normalization of nonuniform incident optical fluence α^N0 as follows:

[α^N]n={[α^N0]n[ϕ^a]n,if  n∈MBreast0,otherwise. (8)

## 4 Study Description and Evaluation Metrics

### 4.1 Computer-Simulation Studies

The proposed method was investigated and evaluated by use of physical measures of image quality and its impact on spectral linear unmixing. Results from the proposed method were compared with those from a general-purpose image contrast enhancement method. To investigate the impact of the proposed method on spectral linear unmixing, ground truth data are required. Accordingly, a realistic numerical breast phantom was generated, and a computer-simulation study was conducted.

#### 4.1.1 Realistic numerical breast phantom

The numerical breast phantom was created using a computational framework for virtual 3D OAT breast imaging trials developed by the authors of Ref. 49. This framework employs an open source tool from the U.S. Food and Drug Administration^50^ with modifications for use in OAT imaging.^49^^,^^51^ The produced numerical breast phantom consists of fat, skin, glandular, nipple, arterial, and venous tissues. The breast shape is a hemisphere with a radius of 60 mm, which is compatible with use of a breast stabilizer cup. The entire phantom was discretized using a uniform 3D Cartesian lattice with a voxel size of 0.25 mm.

#### 4.1.2 Functional, optical, and acoustic properties

Wavelength-dependent optical properties were assigned to each breast tissue by prescribing the composition of each tissue type in terms of total hemoglobin concentration, oxygen saturation, and volume fractions of blood, water, fat, and melanin.^44^ Illumination wavelengths of 757, 800 (the isosbestic point of deoxy- and oxyhemoglobin), and 850 nm were selected from near-infrared-I range (650 to 950 nm) that is commonly used in OAT breast imaging.^5^^,^^7^^,^^9^ While at least two wavelengths are required for the linear unmixing of deoxy- and oxyhemoglobin, additional wavelengths lead to more stable estimates. As data acquisition time increases proportionally to the number of illumination wavelengths used, OAT images at only a few wavelengths can be collected in clinically relevant settings. For this reason, two- and three-wavelength linear unmixing methods were utilized in the numerical studies. Regarding the acoustic properties of the numerical breast phantom, homogeneous speed of sound and no acoustic attenuation were assumed.^52^

#### 4.1.3 Simulation of optoacoustic signals

The optoacoustic signals were simulated in three dimensions using the GPU-accelerated MCXLAB software.^53^^,^^54^ The illumination geometry was configured to mimic LOUISA-3D^5^ (Sec. 2) where laser beams are cylindrically emitted from five linear fiber-optic segments on the surface of an arc-shaped light paddle that rotates around the breast in 20 discrete steps. In the MC simulation, to mimic the beam from each fiber-optic segment, five cone beams with a half-angle of 12.5 deg were employed. Consequently, a total of 500 cone beams were simulated for 20 illumination views. The light source positions were evenly distributed along the linear fiber-optic segments [Fig. 2(a)]. The incident beam direction was specified as perpendicular to the linear segments toward the origin of coordinates [Fig. 3(d)]. The number of photons simulated was $10^{8}$ per cone beam, and the simulation duration was 5 ns. The size of a simulation domain was 340×340×170  voxels with a voxel size of 0.5 mm. Subsequently, the true initial pressure $p_{0}(r,\lambda )$ was computed via elementwise multiplication of the simulated optical fluence and optical absorption coefficient. A Grüneisen parameter Γ=1 was assumed, as is commonly done as constant for soft tissues.^2^^,^^55^

#### 4.1.4 Acoustic wave propagation and data acquisition

Acoustic wave propagation and data acquisition were simulated using the GPU-accelerated k-Wave toolbox.^56^ The measurement geometry was defined as in the LOUISA-3D system:^5^ an arc-shaped probe with 96 transducers collecting pressure data at 320 tomographic views (Fig. 2). A total of 1536 time samples were acquired at virtual transducers with a sampling frequency of 20 MHz. Idealized point-like transducers were assumed. Thermal acoustic noise was modeled as Gaussian noise with zero mean and standard deviation equal to 1% of the maximum signal strength, as was determined based on the *in vivo* breast data.

#### 4.1.5 Image reconstruction and processing

Noisy synthetic data were reconstructed using a GPU-accelerated FBP,^43^ implemented in C++ and CUDA.^38^^,^^57^ The size of the reconstructed volume was 480×480×240  voxels ($120\times 120\times 60\;{\mathrm{mm}}^{3}$). Elapsed time for the image reconstruction was ∼40  s using four NVIDIA GeForce GTX 1080 GPUs. After the image reconstruction, k-means singular value decomposition dictionary learning^58^ was applied to reduce the noise.

For physical image quality evaluation, numerical results from the proposed method were compared with those from contrast limited adaptive histogram equalization (CLAHE),^59^ a method to enhance local contrast that is commonly used in medical images, such as ultrasound images,^60^ mammograms,^61^ and optical microangiographies.^62^ In OAT imaging, CLAHE is employed in a multispectral OAT system (iThera Medical, Germany).^63^ While several implementations of CLAHE are available for one- and two-dimensional images, an extension to 3D images was implemented for use in this study.

For detection and visualization of the blood vasculature, multiscale vessel enhancement filtering^63^^,^^64^ and Otsu thresholding^47^ were applied to the reconstructed initial pressure with no optical fluence normalization, CLAHE, and the proposed method. The vessel enhancement filter, also known as Frangi filter, detects tubular structures based on an eigenvalue analysis of the Hessian matrix of the image at multiple scales.^64^ The thicknesses of the detected structures are controlled through a set of scale parameters. In this work, the parameters with widths ranging from 1 to 5 voxels (0.25 to 1.25 mm) were chosen, as they are representatives of vessel diameters in the breast.^65^

To investigate spectral linear unmixing, molar concentrations of deoxy- and oxyhemoglobin were computed via Eq. (1), from which total hemoglobin concentration $C_{t\mathrm{Hb}}(r)$ and oxygen saturation $sO_{2}(r)$ were subsequently calculated. Results from the two- and three-wavelength linear unmixing methods, with no optical fluence normalization, CLAHE, and the proposed method, were compared.

### 4.2 Studies with Clinical Data

Two clinical data sets corresponding to the right and left breast of a healthy volunteer were acquired by TomoWave Laboratories (Houston, Texas) using LOUISA-3D^5^ at MD Anderson Cancer Center and processed by the authors with institutional review board approval. The breasts were illuminated at a wavelength of 755 nm. The details of the illumination geometry of LOUISA-3D^5^ were given in Sec. 2. Acoustic measurements were collected with ultrawide-band (50 kHz to 6 MHz) ultrasonic transducers of size of $1.1\times 1.1\;{\mathrm{mm}}^{2}$. Additional details of the measurement geometry and image reconstruction were provided in Sec. 4.1. The image reconstruction and processing were conducted identically to those in computer-simulation studies. Experimental results from the proposed method were compared with those from CLAHE.^59^

### 4.3 Evaluation Metrics

#### 4.3.1 Physical measures of image quality

The peak signal-to-noise ratio (PSNR)^66^ and structure similarity (SSIM) index^67^ were calculated. They are defined as

$$\mathrm{PSNR}=10\;\log\;\frac{{\mathrm{MAX}}_{\alpha }^{2}}{\mathrm{MSE}},$$ (9)

and

$$\mathrm{SSIM}(x,y)=\frac{\left(2\mu_{x}\mu_{y}+C_{1})(2\sigma_{xy}+C_{2}\right)}{\left(\mu_{x}^{2}+\mu_{y}^{2}+C_{1})(\sigma_{x}^{2}+\sigma_{y}^{2}+C_{2}\right)}.$$ (10)

In Eq. (9), ${\mathrm{MAX}}_{\alpha }$ is the maximum possible value of voxel brightness (e.g., 255 in 8-bit voxel values), and MSE is the mean squared error with respect to the ground truth, i.e., true $\mu_{a}$ distribution of the blood vessels. In Eq. (10), $\mu_{x}$, $\mu_{y}$, $\sigma_{x}$, $\sigma_{y}$, and $\sigma_{xy}$ are the local means, standard deviations, and covariance of images x and y. Here, x and y correspond to the $\mu_{a}$ estimate, i.e., the reconstructed initial pressure with no normalization, CLAHE, and the proposed method, and the true $\mu_{a}$ distribution of the blood vessels, respectively. $C_{1}={\left(K_{1}{\mathrm{MAX}}_{\alpha }\right)}^{2}$ and $C_{2}={\left(K_{2}{\mathrm{MAX}}_{\alpha }\right)}^{2}$ in Eq. (10) denote stabilization constants, where $K_{1}=0.01$ and $K_{2}=0.03$ are the default values in the Image Processing Toolbox of MATLAB.

#### 4.3.2 Task-based measures of image quality

Blood vessel detectabily and artery/vein classification accuracy were used to evaluate the impact of the proposed method on spectral linear unmixing.

Based on the estimate of total hemoglobin concentration, the blood vessel voxels were detected via multiscale vessel enhancement filtering and Otsu thresholding. The detection performance was assessed using the detectability index DET defined as

DET=N^AVNA+NV×100%, (11)

where $N_{A}$ and $N_{V}$ are the numbers of all voxels corresponding to arteries and veins in the numerical phanton, and ${\hat{N}}_{\mathrm{AV}}$ is the number of voxels correctly detected as vasculature, respectively.

The accuracy of artery/vein classification was assessed under two different scenarios. In the calculation of the classification accuracy index ACC, the vascular structures of the numerical phantom were assumed as known, while in the detection-classification accuracy index (DACC), the vascular structures were estimated from the reconstructed OAT images as explained above. In both scenarios, an oxygenation level of 83.5% was used as the threshold for the classification of arteries and veins. This threshold corresponds to the arithmetic mean of the oxygenation level assigned to arteries (97%) and veins (70%) in the numerical phantom.

Specifically, in the scenario of known vascular structures, true artery rate (TAR), true vein rate (TVR), and classification accuracy index (ACC) were defined as

$$\mathrm{TAR}=\frac{N_{\mathrm{TA}}}{N_{A}}\times 100\%,\;\mathrm{TVR}=\frac{N_{\mathrm{TV}}}{N_{V}}\times 100\%,\;\text{and}\;\mathrm{ACC}=\frac{N_{\mathrm{TA}}+N_{\mathrm{TV}}}{N_{A}+N_{V}}\times 100\%,$$ (12)

where $N_{\mathrm{TA}}$ and $N_{\mathrm{TV}}$ are the numbers of the voxels that are correctly classified as arteries and veins, respectively.

Similarly, in the scenario in which the vascular structures are also estimated from the spectral unmixing of the OAT images, detected true artery rate (DTAR), detected true vein rate (DTVR), and DACC were defined as

DTAR=N^TANA×100%,DTVR=N^TVNV×100%,andDACC=N^TA+N^TVNA+NV×100%, (13)

where ${\hat{N}}_{\mathrm{TA}}$ and ${\hat{N}}_{\mathrm{TV}}$ are the numbers of the voxels that are detected and correctly classified as arteries and veins, respectively.

## 5 Results

### 5.1 Computer-Simulation Studies Results

#### 5.1.1 Physical measures of image quality

**Fig. 9:**
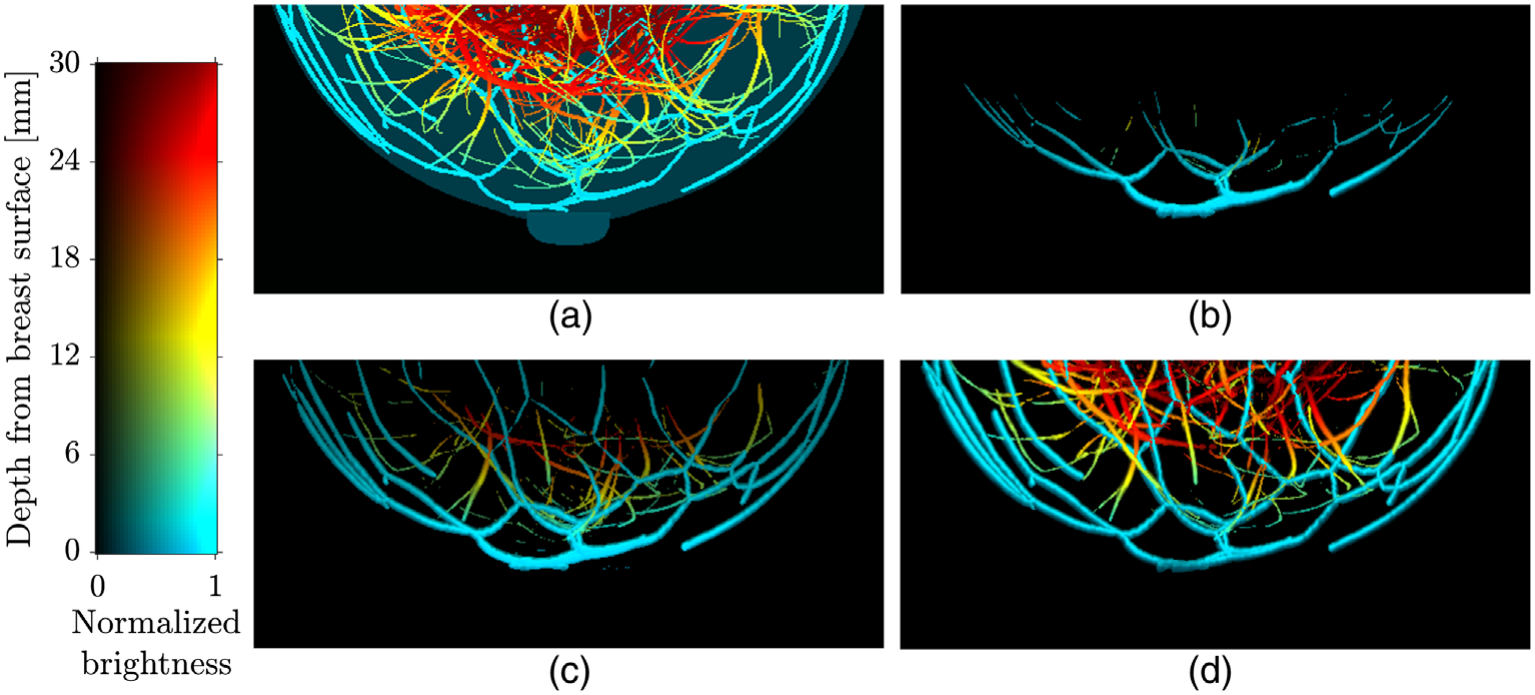
Comparison between distributions of (a) the optical absorption coefficient $\mu_{a}$, (b) initial pressure estimate reconstructed from the noisy measurements, simulated at a wavelength of 800 nm, using FBP with no normalization, and (c) images processed via CLAHE and (d) optical fluence normalization method. The images are presented as MVBP along y axis and color-encoded by depth. A depth range of 0 to 30 mm was visualized. A Jet color map in MATLAB was used to illustrate the breast tissues at different depths.

Figure 9 shows results obtained by applying CLAHE and the proposed optical fluence normalization method to the reconstructed estimate of $p_{0}$. In the results from CLAHE [Fig. 9(c)] and the proposed method [Fig. 9(d)], more structures at depths deeper than 5 mm (green to red color) were revealed compared to the reconstructed initial pressure distribution in Fig. 9(b). The vasculature in the image produced using the proposed optical fluence normalization in Fig. 9(d) is visually more similar to that in the ground truth [Fig. 9(a)] than that produced by CLAHE in Fig. 9(c).

**Fig. 10:**
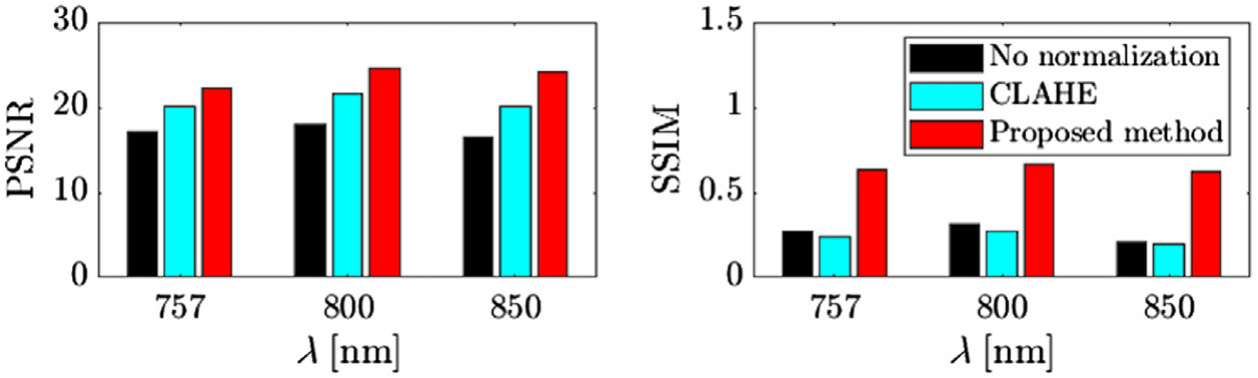
Comparison on PSNR and SSIM between no normalization (black), CLAHE (cyan), and the proposed method (red).

Figure 10 shows PSNR [Eq. (9)] and SSIM [Eq. (10)] comparisons between CLAHE [Fig. 9(c)] and the proposed method [Fig. 9(d)]. As shown in Fig. 10, the results of the proposed method showed higher PSNR and SSIM than those of no normalization and CLAHE for all three wavelengths.

#### 5.1.2 Task-based measures of image quality

**Fig. 11:**
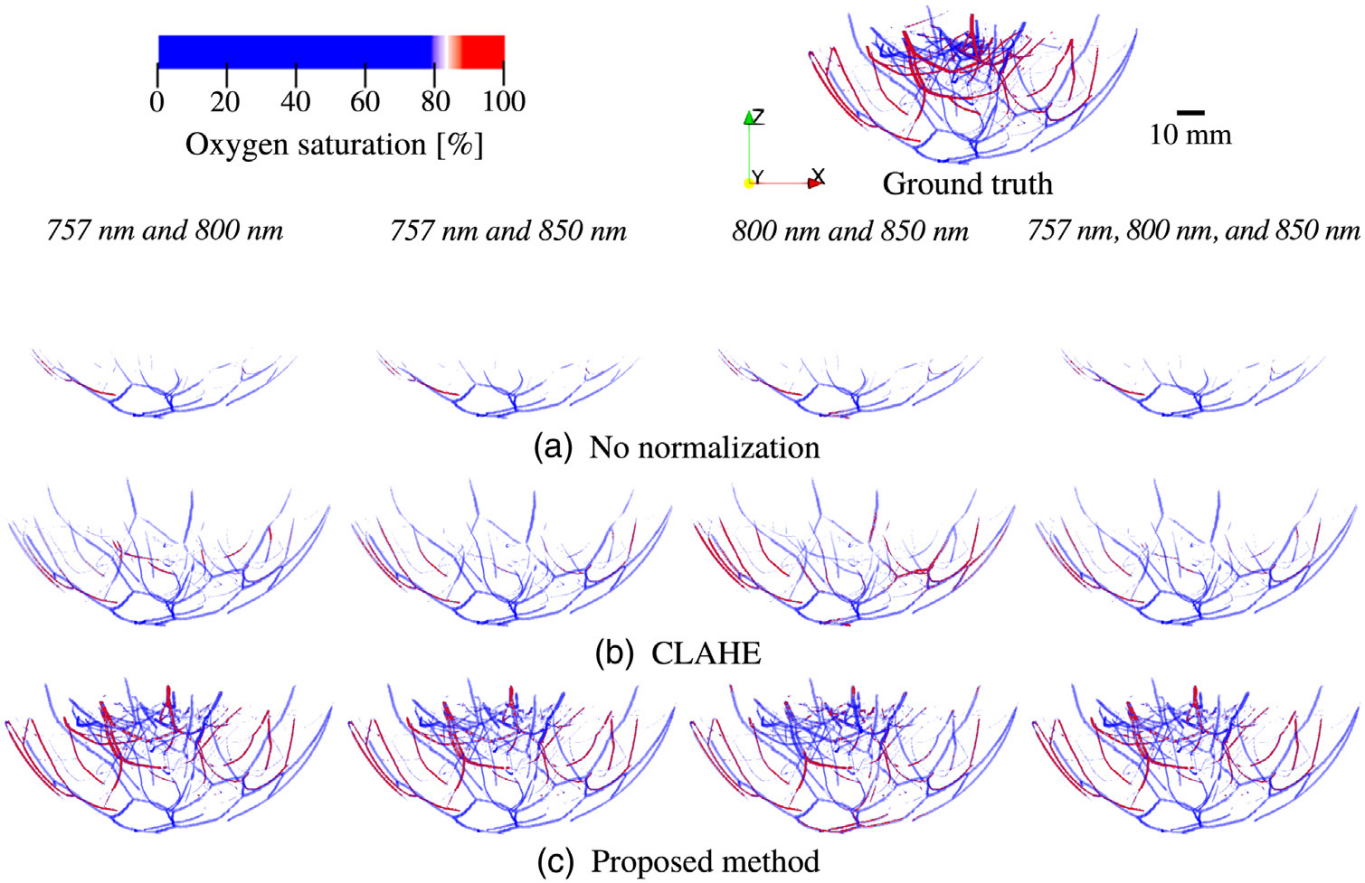
Estimates of vascular blood oxygenation obtained using (a) no optical fluence normalization, (b) CLAHE, and (c) the proposed method. The used wavelength pairs are 757 and 800 nm (first column), 757 and 850 nm (second column), 800 and 850 nm (third column), and 757, 800, and 850 nm (fourth column). The vascular blood oxygenation of the numerical phanton (ground truth) is shown on the top right. Paraview^22^ was used for volume rendering.

Figure 11 shows the detected blood vasculature and the estimated blood oxygenation using two- and three-wavelength linear unmixing methods with no optical fluence normalization [Fig. 11(a)], CLAHE [Fig. 11(b)], and the proposed method [Fig. 11(c)].

With respect to the blood vasculature detection, the majority of the voxels corresponding to the blood vessels were not detected without normalization of the optical fluence [Fig. 11(a)]. Although many of the blood vessel voxels near the breast surface were detected via CLAHE, the voxels in the region deeper than 15 mm [orange to red color in Fig. 9(a)] were not detected [Fig. 11(b)]. The proposed method significantly improved the blood vasculature detectability [Fig. 11(c)]. With respect to estimation of the vascular blood oxygenation, the proposed method [Fig. 11(c)] enhanced the estimation accuracy in regions deeper than 18 mm [orange to red color in Fig. 9(a)] for all choices of wavelengths [Fig. 11(c)]. The results of vascular oxygenation estimation from CLAHE [Fig. 11(b)] were relatively inaccurate regardless of the wavelength pairs and voxel location, compared to the proposed method [Fig. 11(c)].

Table 2 provides detectability (DET), detectability-classification accuracy, and classification accuracy with respect to arteries (TAR and DTAR), veins (TVR and DTVR), and both (ACC and DACC).

**Table 2:** Artery/vein detectability and classification accuracy (%).

λs (nm)	Normalization	DET	TAR	TVR	ACC	DTAR	DTVR	DACC
757, 800	None	12.00	89.81	75.97	81.58	3.62	17.69	11.99
	CLAHE	33.53	72.15	79.16	76.32	16.71	38.83	29.86
	Proposed method	**78.89**	77.88	97.21	**89.38**	66.85	76.69	**72.71**
757, 850	None	11.39	74.78	88.71	83.06	2.78	17.08	11.32
	CLAHE	31.04	62.54	90.82	79.36	16.21	37.12	28.65
	Proposed method	**76.19**	71.62	96.52	**86.43**	58.98	74.38	**68.14**
800, 850	None	11.87	30.41	85.61	63.24	2.88	15.71	10.51
	CLAHE	34.75	25.81	86.07	61.65	16.39	30.37	24.70
	Proposed method	**79.51**	48.66	77.47	**65.79**	37.35	65.33	**53.99**
757, 800, 850	None	11.58	78.35	88.06	84.12	3.07	17.28	11.52
	CLAHE	32.13	64.91	90.03	79.85	16.96	38.05	29.51
	Proposed method	**77.11**	73.12	96.80	**87.21**	61.01	75.24	**69.47**

Note: For each metric and each choice of wavelengths, the entry corresponding to the best performing method is in bold.

**Fig. 12:**
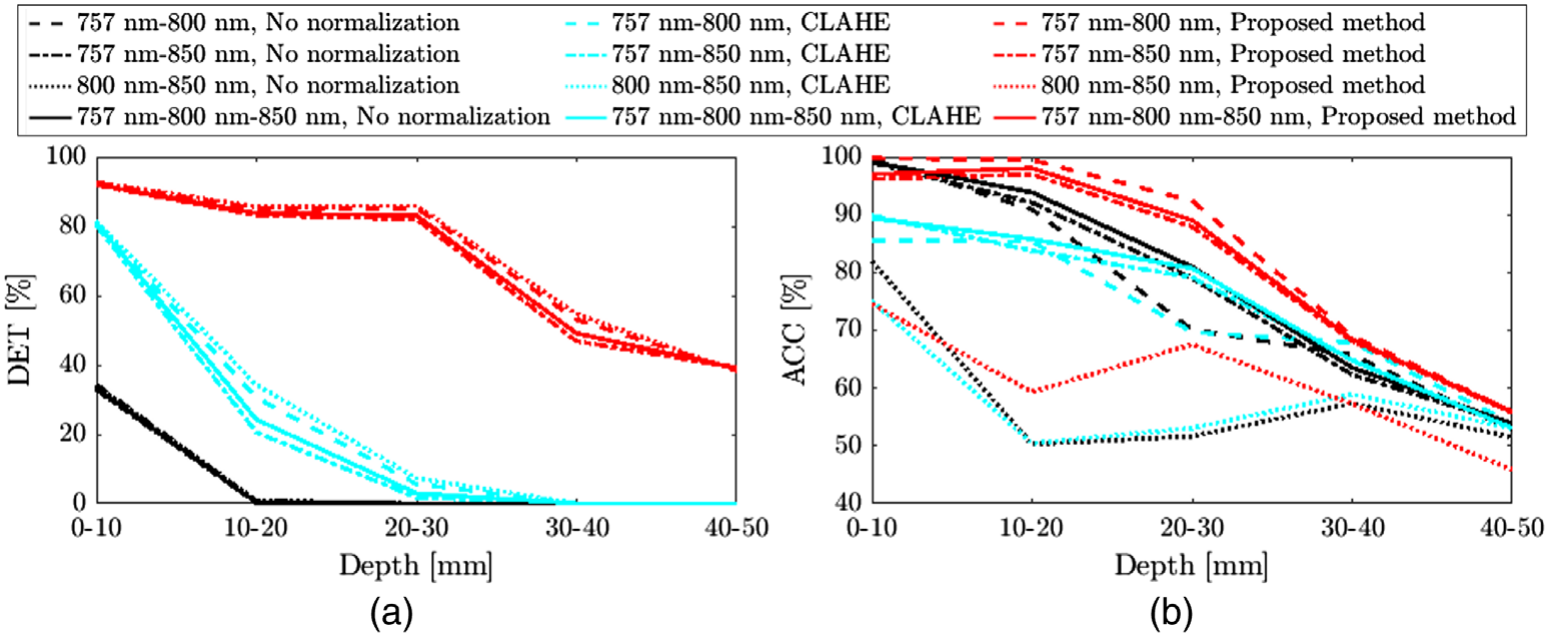
(a) Artery/vein detectability and (b) classification accuracy of no optical fluence normalization (black color), CLAHE (cyan color), and the proposed method (red color), according to 10 mm-depth ranges. The used wavelength pairs are 757 and 800 nm (dashed lines); 757 and 850 nm (dash-dotted lines); 800 and 850 nm (dotted lines); and 757, 800, and 850 nm (solid lines).

As presented in Table 2, the DET of the proposed method was, on average, 6.66 and 2.37 times greater than no optical fluence normalization and CLAHE, respectively. The proposed method showed slightly better ACC compared with the others in Table 2. The proposed method increased the DACC by 5.81 and 2.34 times on average compared to no optical fluence normalization and CLAHE, respectively. The distribution of artery/vein voxels was not uniform with respect to depth. There were more voxels that correspond to the blood vessels (veins in particular) near the surface. Thus, further analysis of the classification accuracy according to depth will be presented henceforward [Fig. 12(b)].

Figure 12 shows (a) vasculature detectability and (b) artery/vein classification accuracy of no optical normalization, CLAHE, and the proposed method as a function of depth. In this analysis, depth was quantized using 5 bins with a width of 10 mm. At all depth ranges, the proposed method [red color in Fig. 12(a)] outperformed the other two [black and cyan colors in Fig. 12(a)] in blood vessel detectability. The artery/vein voxels located deeper than 20 and 30 mm were not detected when no optical fluence normalization [black color in Fig. 12(a)] and CLAHE [cyan color in Fig. 12(a)] were applied, respectively. As shown in Fig. 12(b), in the depth ranges of 10 to 20 mm and 20 to 30 mm, the ACC of the proposed method (red color) was higher than the others (black and cyan colors), and the ACC largely dropped in the results of all three methods at a depth deeper than 30 mm. It is speculated that this is because the strength of the attenuated optoacoustic signals at depths deeper than 30 mm is similar to or lower than that of the noise. The ACC of CLAHE [cyan color in Fig. 12(b)] was either lower or slightly higher, up to 2.53%, than that of no optical fluence normalization [black color in Fig. 12(b)].

#### 5.1.3 Results from Experimental Studies

**Fig. 13:**
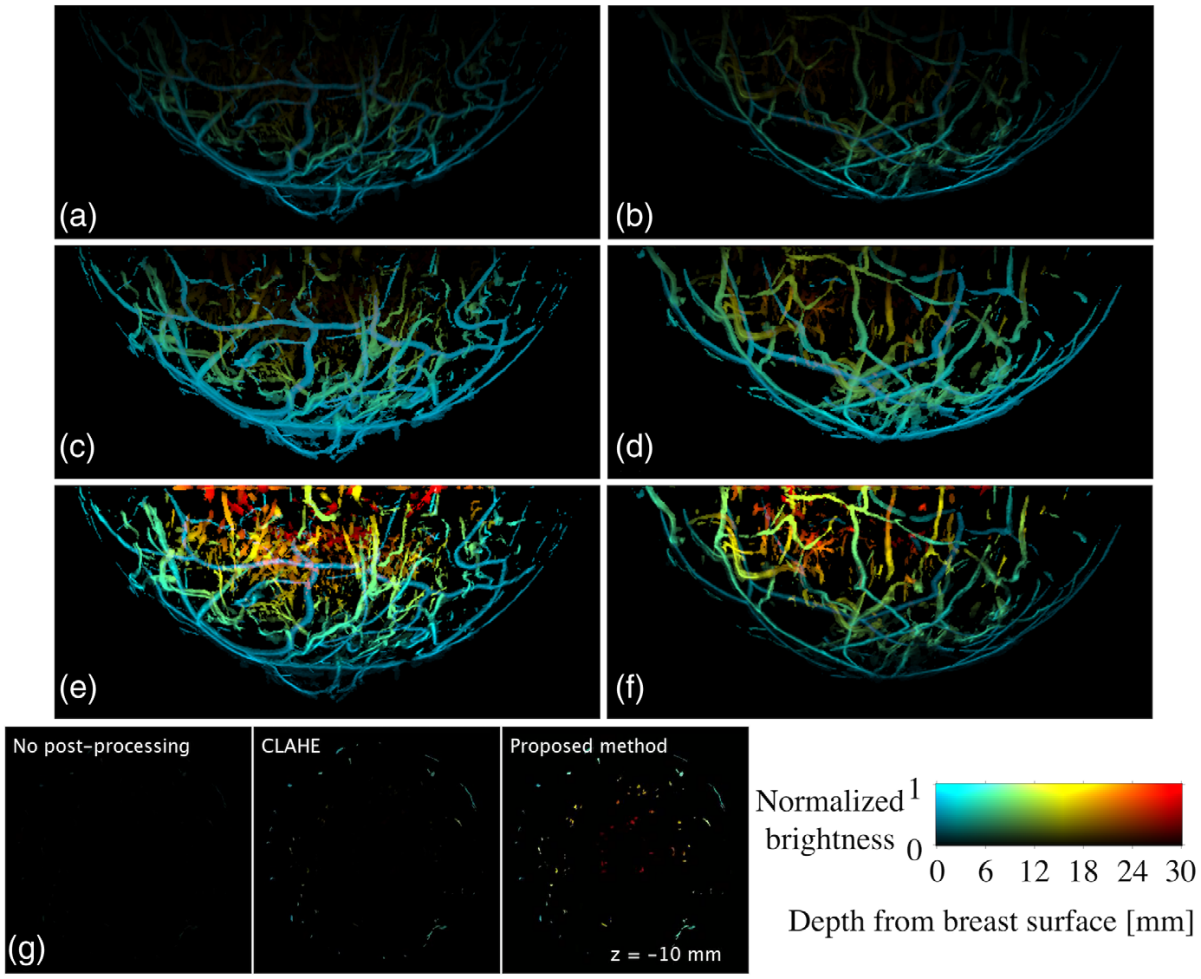
Comparison between reconstructed images with [(a), (b)] no optical fluence normalization, [(c), (d)] CLAHE, and [(e), (f)] the proposed method. The used wavelength was 755 nm. Images in the left column [(a), (c), and (e)] are from the left breast and those in the right column [(b), (d), and (f)] are from the right breast. The images (a) to (f) are presented in MVBP of the entire breast volume along y axis. The still images in panel (g) of Video 1 (Video 1, MP4, 944 kB [URL: https://doi.org/10.1117/1.JBO.27.3.036001.1]) illustrate a z slice (x-y plane) at z=−10  mm. The images were color-encoded by depth using the Jet color map in MATLAB.

Figure 13 shows reconstructed 3D OAT images with no optical fluence normalization [Figs. 13(a) and 13(b)], CLAHE [Figs. 13(c) and 13(d)], and the proposed method [Figs. 13(e) and 13(f)]. A depth range of 0 to 30 mm was visualized. In Fig. 13, the region deeper than 15 mm (orange to red color) is nearly invisible to the human eye in the CLAHE results [Figs. 13(c) and 13(d)] while it is clearly visible in the results of the proposed method [Figs. 13(e) and 13(f)]. Additional visualization of the comparison in Figs. 13(a), 13(c), and 13(e) is provided in Video 1, showing a z-slice (x-y plane) of the breast image at each descretized z location with an increment of 0.25 mm (from −46 to −2.25  mm). In Video 1, the visibility of the blood vessels seated deeper than 15 mm (orange to red color) is consistent with the results in Fig. 13. The effective optical attenuation coefficient estimated from the left breast [Figs. 13(a), 13(c), and 13(e)] was $1\;{\mathrm{cm}}^{-1}$ and that from the right breast [Figs. 13(b), 13(d), and 13(f)] was $0.98\;{\mathrm{cm}}^{-1}$.

## 6 Discussion

In spite of the method’s simplicity, the numerical results demonstrated that the proposed method significantly improved vasculature detectability by compensating for optical attenuation and increased estimation accuracy of the vascular blood oxygenation by mitigating the spectral coloring effect (Figs. 11 and 12, and Table 2). Voxel brightness in the reconstructed estimate of $p_{0}(r,\lambda_{i})$ decreased with depth due to optical attenuation. This resulted in severely underestimating total hemoglobin concentration at depths deeper than 10 mm when applying spectral linear unmixing directly to $p_{0}(r,\lambda_{i})$, rather than $\mu_{a}(r,\lambda_{i})$. On the other hand, in the estimation of oxygen saturation ($C_{{\mathrm{HbO}}_{2}}(r)/C_{t\mathrm{Hb}}(r)$), the effect of the optical attenuation could not be canceled out because of its dependence on wavelengths, i.e., the spectral coloring effect. Thus, the classification accuracy constantly decreased with depth without optical fluence normalization in Fig. 12(b). The proposed method ameliorated such reduction [Fig. 12(b)].

Furthermore, the value of the effective optical attenuation coefficient (Fig. 8), which was estimated from the *in vivo* 3D OAT breast images using the proposed method, correlates well with experimental measurements ($\approx 1\;{\mathrm{cm}}^{-1}$) that were reported in previous studies.^44^^,^^68^^–^^70^ The proposed method is completely measurement-data-driven, therefore, a prior knowledge of the optical properties of the breast tissues, anatomy of the vascular network, and precise characterization of the illumination pattern and incident fluence is not required.

The proposed method was specifically implemented for the 3D OAT breast imaging system presented in Fig. 2. However, the general framework for the normalization of the optical fluence distribution is not limited to breast imaging and to this specific system. For example, the curve fitting for incident optical fluence estimation can be opportunely modified to account for different optical illumination patterns.

Although these studies demonstrated qualitative and quantitative enhancement achieved via use of the proposed method, there remain limitations. First and foremost, the proposed method assumes a constant effective optical attenuation coefficient when estimating the fluence map within the breast. Errors in the estimation of the fluence map due to neglecting spatial variations of effective optical attenuation coefficient may introduce bias in the optical energy absorption estimates.

Besides, to obtain 3D quantitative images of the vascular blood oxygenation from *in vivo* data, further investigations should address acoustic heterogeneity of breast tissue and noise removal (thermal acoustic noise from the medium and thermal noise from ultrasound transducer and electromagnetic interference). Because the proposed method compensates for the depth-dependent optical attenuation by amplifying the image brightness at each voxel in the reconstructed 3D OAT images as a function of depth, existing noise and artifacts are also amplified depending on the depth. Application of the proposed method to images reconstructed using advanced regularization techniques can reduce such noise and artifacts, thus extending imaging depth.

Future directions include investigation of the proposed method to imaging of breasts with benign and malignant lesions and other 3D OAT imaging applications, such as transcranial imaging and small animal imaging (whole or partial body). It is expected that the performance of the proposed method largely depends on the distributions of the optical properties within the target. For example, in whole mouse imaging, hemoglobin-concentrated organs, such as liver, kidneys, and colon, locally occupy a certain extent as bulk. This causes a locally varying imbalance in the optical fluence distribution. In such case, the assumptions of the proposed method are invalid, thus, further investigation is required, including the use of more sophisticated numerical models to estimate the fluence distribution, such as MC photon transport simulation or simplified spherical harmonics approximation of radiative transfer equations.^12^

## 7 Conclusion

In this work, a straightforward physics-based method to normalize optical fluence distributions in 3D OAT breast images was proposed. The method is based on generally accepted assumptions on breast anatomy and optical properties as well as common features of light delivery in existing 3D OAT breast imagers. In the proposed method, both distributions of incident optical fluence and optical attenuation within the breast tissues are estimated solely from the voxel brightness in the reconstructed images, thus, a prior knowledge of the breast and specific geometry of the light-delivery system is not required.

Numerical studies demonstrated that the proposed method—in conjunction with spectral linear unmixing—significantly enhanced blood vasculature detectability and improved estimation accuracy of vascular blood oxygenation down to a depth of 30 mm, when compared with no optical fluence normalization and a general-purpose image contrast enhancement technique called CLAHE. In addition, the proposed method outperformed CLAHE, in terms of PSNR and SSIM. It was also demonstrated that the proposed method can be applied to *in vivo* data. In particular, the effective optical attenuation coefficients estimated from the reconstructed 3D OAT breast images via the proposed method were found to be consistent with those experimentally measured in *in vivo* studies. With further investigations on acoustic heterogeneity, noise removal, and vascular segmentation, the use of the proposed method can potentially achieve 3D *in vivo* functional OAT images of the whole breast.

## Code, Data, and Materials Availability

Data and code are available upon request. Please contact Dr. Park at sp33@illinois.edu.
